# Temporal Trends and Differences in Inpatient Palliative Care Use in Metastatic Penile Cancer Patients

**DOI:** 10.3390/biomedicines13071756

**Published:** 2025-07-18

**Authors:** Carolin Siech, Lukas Scheipner, Andrea Baudo, Mario de Angelis, Letizia Maria Ippolita Jannello, Francesco Di Bello, Fred Saad, Shahrokh F. Shariat, Nicola Longo, Luca Carmignani, Ottavio de Cobelli, Sascha Ahyai, Alberto Briganti, Séverine Banek, Luis A. Kluth, Felix K. H. Chun, Pierre I. Karakiewicz

**Affiliations:** 1 Cancer Prognostics and Health Outcomes Unit, Division of Urology, University of Montréal Health Center, Montréal, QC H2X 0C1, Canada; 2 Goethe University Frankfurt, University Hospital, Department of Urology, 60590 Frankfurt am Main, Germany; 3 Department of Urology, Medical University of Graz, 8010 Graz, Austria; 4 Department of Urology, IRCCS Policlinico San Donato, 20097 Milan, Italy; 5 Unit of Urology, Division of Experimental Oncology, URI, IRCCS Ospedale San Raffaele, 20132 Milan, Italy; 6 Faculty of Medicine and Surgery, Vita-Salute San Raffaele University, 20132 Milan, Italy; 7 Department of Urology, IEO European Institute of Oncology, IRCCS, 20141 Milan, Italy; 8 Department of Urology, Faculty of Medicine, Università degli Studi di Milano, 20122 Milan, Italy; 9 Department of Neurosciences, Science of Reproduction and Odontostomatology, University of Naples Federico II, 80138 Naples, Italy; 10 Department of Urology, Comprehensive Cancer Center, Medical University of Vienna, 1090 Vienna, Austria; 11 Department of Urology, Weill Cornell Medical College, New York, NY 10021, USA; 12 Department of Urology, University of Texas Southwestern Medical Center, Dallas, TX 75390, USA; 13 Hourani Center for Applied Scientific Research, Al-Ahliyya Amman University, Amman 19111, Jordan; 14 Department of Urology, IRCCS Ospedale Galeazzi—Sant’Ambrogio, 20157 Milan, Italy; 15 Department of Oncology and Haemato-Oncology, Università degli Studi di Milano, 20122 Milan, Italy

**Keywords:** palliative supportive care, in-hospital mortality, health disparities, metastatic disease, NIS

## Abstract

**Objectives**: To quantify inpatient palliative care use over time and to test whether patient or hospital characteristics represent determinants of inpatient palliative care use in patients with metastatic penile cancer. **Methods**: Relying on the National Inpatient Sample database (2006–2019), we identified 1017 metastatic penile cancer patients. Estimated annual percentage change analyses and multivariable logistic regression models addressing inpatient palliative care use were fitted. **Results**: Of 1017 metastatic penile cancer patients, 139 (13.7%) received inpatient palliative care. Over time, the proportion of inpatient palliative care use per year increased from 6.5% in 2006 to 17.8% in 2019 (estimated annual percentage change +6.7%; *p* = 0.001). In the multivariable logistic regression models, contemporary study years (odds ratio [OR] 1.80; *p* = 0.003), the presence of bone metastases (OR 1.90; *p* = 0.002) and the presence of brain metastases (OR 2.60; *p* = 0.013) independently predicted higher inpatient palliative care use. Conversely, distant lymph node metastases independently predicted lower inpatient palliative care use (OR 0.58; *p* = 0.022). Finally, hospital admission in the South (OR 2.42; *p* = 0.007) and in the Northeast (OR 2.34; *p* = 0.015) was associated with higher inpatient palliative care use than hospital admission in the Midwest. **Conclusions**: In metastatic penile cancer patients, the proportions of inpatient palliative care use were low but have increased over time. Unfortunately, some geographical regions are more refractory to inpatient palliative care use than others. Finally, specific patient characteristics such as bone metastases and brain metastases represent independent predictors of higher inpatient palliative care use.

## 1. Introduction

Patients with serious illnesses, such as advanced cancer stages, face a high burden of unmet symptoms, coping and communication needs [1]. Specifically, patients may suffer from disease-related physical or mental symptoms, such as dyspnea, pain, anxiety or depression. Palliative care, defined as specialized medical care for people living with a serious illness, might help to relieve symptoms and to decrease psychosocial distress [2,3,4,5]. The assessment of palliative care needs may already begin at the time of diagnosis of a life-limiting medical condition. Benefits of early concurrent palliative care include improved patient as well as caregiver satisfaction, the use of goal-concordant healthcare services and increased hospice use at the end of life [6]. Furthermore, inpatient palliative care may not only improve patients’ quality of life by delivering person-centered as well as interdisciplinary care [1,7,8], but it also represents a favorable indicator of the quality of care [9,10,11]. Therefore, the early and routine integration of inpatient palliative care is a well-established guideline recommendation for advanced cancers, including metastatic penile cancer patients [2,6,12]. According to the recommendations of the National Comprehensive Cancer Network, all patients with cancer should be screened at presentation and reassessed at appropriate intervals or as clinically indicated [2]. However, data regarding palliative care use in metastatic penile cancer patients are rare [13], and contemporary proportions and patterns of inpatient palliative care use are unknown.

We addressed this knowledge gap and hypothesized that guideline-recommended inpatient palliative care use has increased over time and that no regional differences in inpatient palliative care use exist, especially where barriers may prevent patients from accessing inpatient palliative care. Lastly, we tested whether patient or hospital characteristics represent determinants of inpatient palliative care use in metastatic penile cancer patients. To test these hypotheses, we relied on a contemporary population-based cohort of metastatic penile cancer patients within the United States.

## 2. Materials and Methods

### 2.1. Data Source

Relying on discharge data from the National Inpatient Sample from 2006 to 2019, we tested for trends and patterns of inpatient palliative care use in metastatic penile cancer patients. The National Inpatient Sample is a set of longitudinal hospital inpatient databases included in the Healthcare Cost and Utilization Project and formed by the Agency for Healthcare Research and Quality through a federal–state partnership [14]. The National Inpatient Sample selects approximately 20% of the discharges from United States community non-rehabilitation hospitals, excluding long-term acute care hospitals [14]. Prior to 2012, the National Inpatient Sample was a stratified probability sample of hospitals with sampling probabilities proportional to the number of United States community hospitals in each stratum. Every selected hospital reported all their discharges. Starting with the 2012 National Inpatient Sample, a systematic sample of 20% of the discharges was drawn from all hospitals. The new systematic self-weighted sample design ensures that the sample is representative of the population regarding hospital and patient factors [14].

All diagnoses and procedures were coded using the International Classification of Diseases (ICD) 9th revision Clinical Modification (ICD-9-CM) and the ICD 10th revision Clinical Modification (ICD-10-CM), as well as the ICD 10th revision Procedure Coding System (ICD-10-PCS).

### 2.2. Study Population

We focused on patients aged ≥ 18 years with a diagnosis of penile cancer (ICD-9-CM codes 187.1–187.4 and ICD-10-CM codes C60, C60.1, C60.2, C60.8, C60.9) and a secondary diagnosis of metastatic disease (ICD-9-CM codes 196.0–196.4, 196.7–196.9, 197.x, 198.x and ICD-10-CM codes C77.0-C77.3, C77.6-C77.9, C78.x, C79.x). Since local invasion is common in patients with advanced penile cancer and does not fulfill the criteria for distant metastasis, patients with exclusive secondary malignant neoplasms of the genital (ICD-9-CM code 198.82 and ICD-10-CM code C79.82) or urinary organs (ICD-9-CM code 198.1 and ICD-10-CM code C79.1x) were excluded.

### 2.3. Definition of Variables for Analyses

According to prior validation studies, the use of inpatient palliative care, the primary end point of the study, was coded using ICD-9-CM code V66.7 and ICD-10-CM code Z51.5 [15,16,17,18,19]. Covariables consisted of age at admission (years, continuously coded), race/ethnicity (African American vs. others vs. Caucasian), insurance status (Medicaid vs. private vs. other vs. Medicare), hospital region (Northeast vs. West vs. Midwest vs. South), teaching hospital status (teaching vs. non-teaching) and hospital size (small [<200 beds] vs. medium [200–399 beds] vs. large [≥400 beds]). Hospital regions are based on the United States census divisions: Northeast summarizes New England and the Middle Atlantic; Midwest includes the East and West North Central; South includes the South Atlantic, East and West South Central; and West incorporates the Mountain and Pacific census divisions [14]. Additional risk variables consisted of the number (multiple sites vs. single site) and the location of metastatic sites (regional lymph nodes [yes vs. no], distant lymph nodes [yes vs. no], lung [yes vs. no], bone [yes vs. no], liver [yes vs. no] and brain [yes vs. no]). Year of admission was dichotomized in year intervals (2015–2019 vs. 2006–2014) for univariable and multivariable analyses.

### 2.4. Statistical Analyses

To assess contemporary patterns in inpatient palliative care use, three analytical steps were performed. First, descriptive characteristics were tabulated. The median, first and third interquartile were recorded for continuously coded variables, and the Wilcoxon rank sum test examined the statistical significance of medians’ differences. Frequencies and proportions were recorded for categorical variables, and Pearson’s chi-squared test assessed the statistical significance in proportions’ differences. Second, estimated annual percentage changes for inpatient palliative care use were tested with least squares linear regression. Third, univariable and multivariable logistic regression models were used to test for predictors of inpatient palliative care use. All multivariable analyses were fitted after adjustment for clustering at the hospital level, relying on a generalized estimating equation methodology [18,19,20]. Adjustment variables in the multivariable analyses consisted of statistically significant and clinically meaningful patient and hospital characteristics in the univariable analyses.

Analyses and reporting followed the National Inpatient Sample reporting rules [14]. For sample sizes of less than eleven patients, counts and associated proportions were reported as less than eleven. Due to the anonymously coded design of the National Inpatient Sample, study-specific ethics approval was not required. All tests were two-sided, with a significance level set at *p* < 0.05. The R software environment was used for statistical computing and graphics (R version 4.3.2; R Foundation for Statistical Computing, Vienna, Austria) [21].

## 3. Results

### 3.1. Descriptive Characteristics

Overall, we identified 1017 metastatic penile cancer patients within the National Inpatient Sample (2016–2019; Table 1). Of these, 139 (13.7%) received inpatient palliative care. The proportion of inpatient palliative care use was the lowest in the Midwest (8.3%) and the highest in the Northeast (16.4%). Inpatient palliative care patients were more frequently treated in contemporary study years (57.6 vs. 42.1%; *p* < 0.001) and more frequently exhibited multiple metastatic sites (44.6 vs. 34.6%; *p* = 0.023), bone metastases (38.8 vs. 22.7%; *p* < 0.001) and brain metastases (7.9 vs. 3.1%; *p* = 0.005). Conversely, patients receiving inpatient palliative care less frequently exhibited distant lymph node metastases (15.8 vs. 23.7%; *p* = 0.040). The median length of stay (6.0 vs. 4.0 days; *p* < 0.001) and in-hospital mortality (24.5 vs. 4.9%; *p* < 0.001) were both higher in patients with inpatient palliative care use.

### 3.2. Temporal Trends of Inpatient Palliative Care Use and Inpatient Mortality

**Figure 1:**
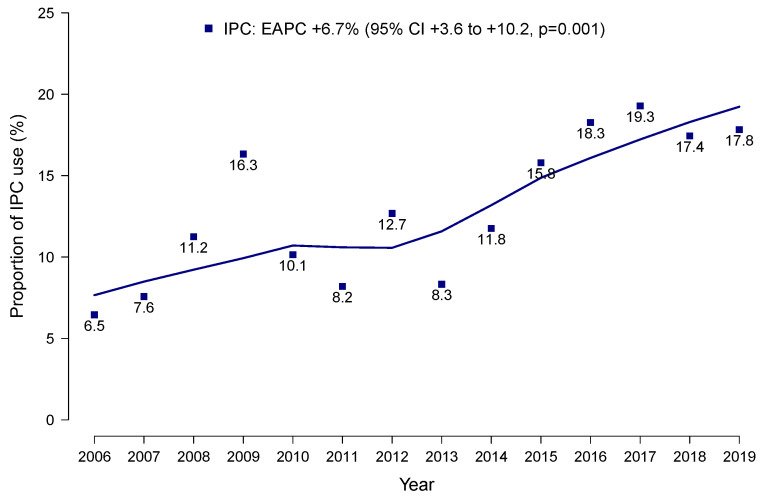
The proportion of patients using inpatient palliative care per year, as well as the estimated annual percentage change in inpatient palliative care use in metastatic penile cancer patients within the National Inpatient Sample from 2006 to 2019. Abbreviations: CI = confidence interval; EAPC = estimated annual percentage change; IPC = inpatient palliative care.

The proportion of inpatient palliative care use in metastatic penile cancer patients increased from 6.5% in 2006 to 17.8% in 2019 (estimated annual percentage change +6.7%; 95% CI: +3.6 to +10.2%; *p* = 0.001; Figure 1). Conversely, no difference in in-hospital mortality was recorded over time (estimated annual percentage change −1.6%, 95% confidence interval −8.1 to 5.0%; *p* = 0.630). In terms of hospital region, inpatient palliative care use increased from 13.3% to 20.0% in the South, from <0.5 to 20.0% in the Northeast, from <0.5 to 19.0% in the West and from <0.5 to 10.0% in the Midwest.

### 3.3. The Associations Between Hospital Characteristics and Metastatic Patterns and Inpatient Palliative Care Use

In metastatic penile cancer patients, contemporary study years (multivariable odds ratio 1.80, 95% confidence interval 1.22–2.66; *p* = 0.003), the presence of bone metastases (odds ratio 1.90, 95% confidence interval 1.27–2.85; *p* = 0.002) and the presence of brain metastases (odds ratio 2.60, 95% confidence interval 1.23–5.53; *p* = 0.013) independently predicted higher inpatient palliative care use (Table 2). Conversely, distant lymph node metastases were an independent predictor of lower inpatient palliative care use (odds ratio 0.58, 95% confidence interval 0.37–0.92; *p* = 0.022). Moreover, hospital admissions in the South (odds ratio 2.42, 95% confidence interval 1.28–4.59; *p* = 0.007) and in the Northeast (odds ratio 2.34, 95% confidence interval 1.18–4.64; *p* = 0.015) were associated with higher inpatient palliative care use than admission to hospitals in the Midwest.

## 4. Discussion

Multiple randomized controlled trials demonstrated positive effects of the early and routine implementation of palliative care into standard oncological care [7,22,23,24]. Among others, the benefits of the concurrent use of palliative care include improved physical and mental well-being and improved patient and caregiver satisfaction, as well as improved quality of life for both the patient and their family [1,9,10,11]. Therefore, inpatient palliative care represents a well-established guideline recommendation for advanced cancers, including metastatic penile cancer patients [2,12]. However, few studies have addressed palliative care use in patients with genitourinary cancers [1,4,13,18,19,20]. In particular, data regarding palliative care use in metastatic penile cancer patients are rare [13]. Relying on a contemporary population-based cohort of metastatic penile cancer patients within the National Inpatient Sample (2006–2019), we tested for temporal trends and patterns of inpatient palliative care use. We made several noteworthy observations.

First, penile cancer represents a rare primary with a poor prognosis [25]. Within penile cancer of all stages, metastatic penile cancer represents an even rarer entity [13,26]. Specifically, Wenzel et al. reported that only 142 (3.4%) of penile cancer patients exhibited a metastatic stage within the Surveillance, Epidemiology and End Results database between 2004 and 2016 [26]. Similarly, Davaro et al. could only identify 297 metastatic penile cancer patients within the National Cancer Database between 2004 and 2015 [13]. In the current study, we identified 1017 metastatic penile cancer patients over a period of fourteen years (National Inpatient Sample 2006–2019). In consequence, large-scale population-based datasets such as the National Inpatient Sample are essential to study rare events, such as metastatic penile cancer with concurrent inpatient palliative care use, as was done in the current study.

Second, in metastatic penile cancer patients, the overall proportion of inpatient palliative care use was only 13.7%. Over time, the annual proportion of inpatient palliative care use increased from 6.5% to 17.8% between 2006 and 2019 (estimated annual percentage change +6.7%; *p* = 0.001). The currently observed proportion of inpatient palliative care use was comparable to the proportion of palliative care use of 17.6% reported in a historical series of metastatic penile cancer patients within the National Cancer Database (2004–2015) [13]. In contrast to Davaro et al., describing a stable proportion of inpatient palliative care use, we observed an increase in the inpatient palliative care use over time [13]. This observation is encouraging and may be driven by multiple factors, such as greater awareness, the training of healthcare professionals and structural support. The introduction of specific palliative care clinical practice guidelines played a major role in standardizing palliative care practices in oncology and encouraging earlier referrals [2,6]. The increase in inpatient palliative care use might imply an improvement in patient care, as well as stronger adherence to guideline recommendations [2,6,12]. However, the current proportion of inpatient palliative care use is lower than the inpatient palliative care use described by Patel et al. (20.0%) and Han et al. (28.4%) in advanced kidney and metastatic bladder cancer patients, respectively [27,28]. Additionally, the present inpatient palliative care use in metastatic penile cancer patients is significantly lower than the reference standard reported in metastatic lung cancer (50.1%) [29] or metastatic breast cancer (21.9%) [30], where the proportions of inpatient palliative care use are among the highest. Therefore, clinicians, as well as oncology nurses, caring for metastatic penile cancer patients should be further sensitized with the intention of increasing and ideally reaching the proportions of inpatient palliative care use recorded in patients with other metastatic primaries, such as metastatic lung cancer.

Third, we identified important regional differences in inpatient palliative care use among metastatic penile cancer patients. Specifically, the proportion of inpatient palliative care use in hospitals in the South and in the Northeast was more than two-fold higher than in hospitals in the Midwest (14.7 and 16.4 vs. 8.3%; multivariable odds ratio 2.42 and 2.34). Regional differences in inpatient palliative care use may be attributed to a variety of factors. First, regional differences may be explained by the palliative care facilities themselves, which may have different outreach programs in different regions. Moreover, the critical shortage of trained palliative care specialists is more severe in certain regions, such as rural areas, compared to others. Second, organizational structures vary between different regions like the Midwest and the South. For example, in the western states, retirement and palliative care is often provided by large organizations, including large hotel corporations with extensive commercial outreach. The presence of these organizations may impact access to inpatient palliative care. Additionally, providers in certain regions may be more familiar with supportive or palliative care. Therefore, variations in the training and awareness of clinicians may influence the referral culture regarding specialized palliative care. Moreover, cultural and religious beliefs can shape patient preferences regarding end-of-life care, contributing to regional variations in inpatient palliative care in the United States. However, the SEER database does not provide such detailed information. Therefore, the proposed explanations are preliminary at best. Future research, relying on qualitative (e.g., interviews, focus groups) or mixed-methods studies (qualitative and quantitative approaches), is necessary to explore local barriers, practices or attitudes. Nonetheless, the existence of differences in inpatient palliative care use between different regions of the United States is worrisome. Such differences should ideally not exist. Their presence should motivate all health professionals to sensitize their peers about the availability of inpatient palliative care in metastatic penile cancer patients. Moreover, regional differences in inpatient palliative care use should motivate all health professionals to identify differences in the outreach programs of palliative care facilities and to implement palliative care as an important longitudinal component in all medical training curricula across the United States.

Fourth, we identified important differences in inpatient palliative care use according to the location of metastases. Certain metastatic sites, such as the bone (38.8 vs. 22.7%) and brain (7.9 vs. 3.1%), were more frequently recorded in patients who benefited from inpatient palliative care and were independent predictors of inpatient palliative care use (bone odds ratio 1.90; brain odds ratio 2.60). Conversely, the presence of distant lymph node metastases was less frequently documented in metastatic penile cancer patients that benefited from inpatient palliative care (15.8 vs. 23.7%) and independently predicted lower use (odds ratio 0.58). These observations validate the classic clinical perception of disease severity where certain metastatic sites, such as the bone or brain, are perceived as more worrisome. Therefore, local faculties recognize that there are fewer treatment options and request inpatient palliative care more frequently for such patients. Conversely, other sites, such as lymph node metastases, do not carry the same worrisome connotations among clinicians. The devaluation of such distinction between worrisome and less worrisome metastatic sites has never been tested in metastatic penile cancer patients. In consequence, inpatient palliative care should be recommended to all metastatic penile cancer patients regardless of the specific metastatic distribution, unless otherwise clinically indicated. Such an approach, where inpatient palliative care is more universally requested, instead of being used in a systematic fashion, should avoid barriers to inpatient palliative care use in specific patient subgroups that may equally benefit from inpatient palliative care relative to other metastatic penile cancer patients.

Fifth, in the present analyses, the median total hospital charges were higher in patients receiving palliative care compared to their counterparts without palliative care use. The higher hospital charges in patients receiving palliative care may be explained in multiple ways. First, palliative care use was associated with a longer hospital stay (median 6.0 vs. 4.0 days). Second, patients receiving palliative care more frequently exhibited multiple metastatic sites (44.6 vs. 34.6%). A higher number of metastatic sites may lead to more diverse palliative care demands, requiring more complex symptom management relative to those with single-site metastases. Lastly, patients receiving palliative care may have multiple comorbidities, increasing the need for specialist consultations, advanced diagnostics and interventions. The observed statistically non-significant differences in the total hospital charges appear to primarily reflect the culture of referral to palliative care at a late stage in penile cancer and should not discourage from referring patients to specialized palliative care, as it is associated with numerous benefits, particularly when initiated early [7,22,23,24].

Taken together, our observations indicate low inpatient palliative care use that is gradually increasing. It is worrisome that, in some regions, the proportions of inpatient palliative care use are significantly lower than in others. Finally, there appear to be specific patient profiles where inpatient palliative care use is systematically higher than in others. Until proven otherwise, the above regional and patient profile differences should ideally not dictate inpatient palliative care use, so as to avoid barriers to inpatient palliative care access in some regions or in some metastatic penile cancer individuals.

Despite the growing recognition of its benefits, the comprehensive implementation of inpatient palliative care in the United States remains limited. Several actions should be considered to address these challenges. To achieve greater awareness among clinicians, the implementation of routine screening for the burden of unmet symptoms, coping and communication needs at initial diagnosis, as well as at every hospital admission, in patients with metastatic-stage penile cancer could represent a first step. Additionally, handouts and specific information material addressing the availability of inpatient palliative care to share with patients and their families could represent a second step. The critical shortage of trained palliative care specialists, particularly in rural areas, highlights the need for expanded workforce development programs and incentives to enter the field [31,32]. It is necessary to improve training opportunities, as well as the attractiveness of the medical specialty, for all healthcare providers to reduce regional disparities. Longitudinal programs integrated into all medical curricula, as well as annual workshops and targeted training programs, could dispel the misconceptions equating palliative care with hospice [33]. Moreover, reforms to current reimbursement models are essential to better support the time- and labor-intensive nature of palliative care across the United States [34].

The present study is not devoid of limitations. First, we used a large retrospective database with its inherent limitations. Despite systematic adjustment for biases and confounders, the potential for selection and reporting biases remained. This limitation is in common with all studies relying on retrospective databases, such as the Surveillance, Epidemiology and End Results database or the National Cancer Database [13,26,35,36,37]. Second, we relied on previously established and validated methodologies based on both ICD-9 and ICD-10 codes for the identification of inpatient palliative care use [15,16,17,18,19]. However, inpatient palliative care definitions, as well as methodological approaches, may differ [38]. In consequence, other approaches may result in proportions of inpatient palliative care use that are not directly comparable. Third, palliative care can be delivered in inpatient, outpatient or home-based settings [1,9]. Since the National Inpatient Sample exclusively provides inpatient data, such as inpatient palliative care and inpatient mortality, studies relying on the National Inpatient Sample may underestimate overall palliative care use including outpatient and inpatient care. Finally, there is only a limited amount of detail available in the National Inpatient Sample. The National Inpatient Sample does not provide detailed information about the palliative care interventions and measures applied, such as pain relief or anxiety management. Granular information regarding the clinical or pathological tumor stage (Tstage) at palliative care referral, as well as the histopathological subtype of penile cancer, is not available in the currently used version of the National Inpatient Sample. Additionally, other covariables, such as marital status and social support, that may have influenced medical decision-making, including inpatient palliative care use, are not available in the National Inpatient Sample. However, we relied on multivariable adjustment with clinically meaningful characteristics offered by the National Inpatient Sample.

## 5. Conclusions

In metastatic penile cancer patients, the proportion of inpatient palliative care use was low but has increased over time. Unfortunately, some geographical regions are more refractory to inpatient palliative care use than others. Finally, specific patient characteristics such as bone metastases, brain metastases and multiple metastatic sites represent independent predictors of higher inpatient palliative care use.

## Figures and Tables

**Table 1:** Baseline characteristics of 1017 metastatic penile cancer patients, stratified according to inpatient palliative care use.

Characteristic		Overall, n = 1017	Inpatient Palliative Care, n = 139 (13.7%)	No Inpatient Palliative Care, n = 878 (86.3%)	*p*-Value
Age at admission (in years) ^1^		64.0 (53.0–74.0)	62.0 (52.0–73.0)	65.0 (53.0–74.0)	0.485 ^3^
Race/ethnicity ^2^	Caucasian	549 (54.0%)	75 (54.0%)	474 (54.0%)	0.846 ^4^
	African American	118 (11.6%)	18 (12.9%)	100 (11.4%)	
	Other	350 (34.4%)	46 (33.1%)	304 (34.6%)	
Insurance status ^2^	Medicare	468 (46.0%)	51 (36.7%)	417 (47.5%)	0.106 ^4^
	Medicaid	187 (18.4%)	29 (20.9%)	158 (18.0%)	
	Private	271 (26.6%)	46 (33.1%)	225 (25.6%)	
	Other	91 (9.0%)	13 (9.3%)	78 (8.9%)	
Length of stay (in days) ^1^		4.0 (3.0–8.0)	6.0 (3.0–10.5)	4.0 (3.0–8.0)	**<0.001** ^3^
Total hospital charges (in USD) ^1^		37,868.6 (19,720.6–68,045.2)	42,496.4 (19,069.5–90,171.6)	36,957.3 (19,968.3–65,673.0)	0.163 ^3^
Hospital region ^2^	South	389 (38.2%)	57 (41.0%)	332 (37.8%)	0.098 ^4^
	Northeast	250 (24.6%)	41 (29.5%)	209 (23.8%)	
	West	198 (19.5%)	26 (18.7%)	172 (19.6%)	
	Midwest	180 (17.7%)	15 (10.8%)	165 (18.8%)	
Non-teaching hospital status ^2^		289 (28.4%)	33 (23.7%)	256 (29.2%)	0.188 ^4^
Hospital size ^2^	Large (≥400 beds)	669 (65.9%)	98 (70.5%)	571 (65.2%)	0.385 ^4^
	Medium (200–399 beds)	216 (21.3%)	29 (20.9%)	187 (21.3%)	
	Small (<200 beds)	130 (12.8%)	12 (8.6%)	118 (13.5%)	
Contemporary year at admission ^2^	2015–2019	450 (44.2%)	80 (57.6%)	370 (42.1%)	**<0.001** ^4^
Number of metastatic sites ^2^	Single site	651 (64.0%)	77 (55.4%)	574 (65.4%)	**0.023** ^4^
	Multiple sites	366 (36.0%)	62 (44.6%)	304 (34.6%)	
Location of metastasis ^2^	Regional lymph nodes	255 (25.1%)	31 (22.3%)	224 (25.5%)	0.417 ^4^
	Distant lymph nodes	230 (22.6%)	22 (15.8%)	208 (23.7%)	**0.040** ^4^
	Lung	355 (34.9%)	45 (32.4%)	310 (35.3%)	0.500 ^4^
	Bone	253 (24.9%)	54 (38.8%)	199 (22.7%)	**<0.001** ^4^
	Liver	89 (8.8%)	18 (12.9%)	71 (8.1%)	0.059 ^4^
	Brain	38 (3.7%)	11 (7.9%)	27 (3.1%)	**0.005** ^4^
Primary diagnosis at admission ^2^	Cancer-related disorders	395 (38.8%)	69 (59.6%)	326 (37.1%)	**<0.001** ^4^
	Infections	202 (19.9%)	41 (29.5%)	161 (18.3%)	
	Other disorders	420 (41.3%)	29 (20.9%)	391 (44.6%)	
Do not resuscitate status ^2^		124 (12.2%)	62 (44.6%)	62 (7.1%)	**<0.001** ^4^
In-hospital death ^2^		77 (7.6%)	34 (24.5%)	43 (4.9%)	**<0.001** ^4^

^1^ Median (interquartile range: Q1–Q3); ^2^ n (%); ^3^ Wilcoxon rank sum test (examined the statistical significance of medians’ differences); ^4^ Pearson’s chi-squared test (examined the statistical significance of proportions’ differences). Bold highlights statistically significant *p*-value.

**Table 2:** Univariable and multivariable logistic regression models addressing the use of inpatient palliative care in metastatic penile cancer patients after adjustment for clustering at the hospital level using a generalized estimating equation methodology. The use of inpatient palliative care was defined as 1. Conversely, no use of inpatient palliative care was defined as 0.

	Univariable		Multivariable *	
Outcome of Interest: *Inpatient Palliative Care Use*	Odds Ratio (95% Confidence Interval)	*p*-Value	Odds Ratio (95% Confidence Interval)	*p*-Value
Hospital region (reference: Midwest)				
West	1.69 (0.85, 3.37)	0.137	1.89 (0.94, 3.82)	0.076
Northeast	**2.17** (1.11, 4.23)	**0.024**	**2.34** (1.18, 4.64)	**0.015**
South	**2.05** (1.10, 3.84)	**0.024**	**2.42** (1.28, 4.59)	**0.007**
Non-teaching hospital status (reference: Teaching hospital status)	0.72 (0.47, 1.12)	0.143	–	–
Hospital size (reference: Large)				
Medium	0.85 (0.53, 1.36)	0.498	–	–
Small	0.58 (0.31, 1.09)	0.092	–	–
Location of metastatic site				
Regional lymph nodes (reference: No)	0.88 (0.59, 1.32)	0.541	–	–
Distant lymph nodes (reference: No)	**0.60** (0.39, 0.94)	**0.024**	**0.58** (0.37, 0.92)	**0.022**
Lung (reference: No)	0.89 (0.61, 1.30)	0.546	–	–
Bone (reference: No)	**2.03** (1.39, 2.97)	**<0.001**	**1.90** (1.27, 2.85)	**0.002**
Liver (reference: No)	1.61 (0.95, 2.72)	0.075	–	–
Brain (reference: No)	**2.72** (1.32, 5.62)	**0.007**	**2.60** (1.23, 5.53)	**0.013**
Multiple metastatic sites (reference: Single metastatic site)	**1.53** (1.06, 2.20)	**0.022**	1.32 (0.88, 1.99)	0.175
Year interval 2015–2019 (reference: 2006–2014)	**1.72** (1.17, 2.52)	**0.006**	**1.80** (1.22, 2.66)	**0.003**

* adjusted for hospital region, metastatic site and year interval. Bold highlights statistically significant odds ratio and corresponding *p*-value.

## Data Availability

The data used in this study (National Inpatient Sample 2006–2019) are owned by the Healthcare Cost and Utilization Project and are publicly available after purchase (https://www.distributor.hcup-us.ahrq.gov/Databases.aspx, accessed on 9 July 2025). The authors confirm that they did not have any special access privileges that others would not have.

## Abbreviations

The following abbreviations are used in this manuscript:

ICD	International Classification of Diseases
ICD-9-CM	ICD 9th Revision Clinical Modification
ICD-10-CM	ICD 10th Revision Clinical Modification
ICD-10-PCS	ICD 10th Revision Procedure Coding System
